# Supplementary material for: Event-triggered STED imaging
Source: Nat Methods. 2022 Sep 8;19(10):1268–75. doi: 10.1038/s41592-022-01588-y (PMC9550628; doi:10.1038/s41592-022-01588-y)
Supplement: Supplementary file 1 — Supplementary Notes 1–10, Supplementary Figs. 1–6 and Supplementary Tables 1–3 [file 41592_2022_1588_MOESM1_ESM.pdf]

nature portfolio

https://doi.org/10.1038/s41592-022-01588-y

Supplementary information

Event-triggered STED imaging

In the format provided by the authors and unedited

Supplementary information

Event-triggered STED imaging

Jonatan Alvelid[1], Martina Damenti[1], Chiara Sgattoni[1] and Ilaria Testa[1]

[1]*Department of Applied Physics and Science for Life Laboratory, KTH Royal Institute of Technology, 100 44 Stockholm, Sweden*

Corresponding author: Ilaria Testa

Email: ilaria.testa@scilifelab.se

List of content

1. **Supplementary Notes**
2. **Supplementary Figures**
3. **Supplementary Tables**

List of Supplementary Notes

1. Real-time rapid signal spikes detection pipeline
2. Real-time rising signal detection pipeline
3. Real-time vesicle proximity detection pipeline
4. Software implementation and generic control widget
5. General event-triggered control implementation
6. Example etSTED experiments
7. Post-acquisition true/false rapid signal spike event decision
8. Temporal characterization of real-time analysis pipelines and etSTED experiments
9. Manual STED timelapses of synaptotagmin-1
10. Microscope components

List of Supplementary Figures

1. Optimized rapid signal spikes event detection analysis pipeline.
2. etSTED setup.
3. Post-acquisition true/false detected event decision.
4. Temporal characterization for etSTED experiments using the *rapid_signal_spikes* analysis pipeline.
5. etSTED experiment in neurons with calcium imaging (Oregon Green 488 BAPTA-1) and STED timelapse imaging of actin (SiR-actin).
6. etSTED experiment in neurons with calcium imaging (Oregon Green 488 BAPTA-1) and STED timelapse imaging of microtubules (SiR-tubulin).

List of Supplementary Tables

1. etSTED analysis pipeline parameters for the data presented in the figures.
2. etSTED sample and image acquisition parameters for widefield and STED imaging for the data presented in the figures.
3. Runtimes of etSTED analysis pipelines for CPU/GPU application and various image sizes.

Supplementary Notes

Supplementary Note 1. Real-time rapid signal spikes detection pipeline

The first analysis pipeline used in this work is developed and optimised for detection of calcium activity spikes in hippocampal neurons in widefield images of Oregon Green 488 BAPTA-1 as well as rapid intensity spikes in HeLa cells in widefield images of CD63-pHluorin, both with a pixel size of 100 nm. The pipeline is used in the etSTED widget as a separate python function and uses the current and previous widefield images as input, and outputs the ratiometrically brightest detected event coordinates. The pipeline is schematically shown in Supplementary Fig. 1 and is available at https://github.com/jonatanalvelid/ImSwitch-etSTED/blob/master/smartsted/analysis_pipelines/bapta_calcium_spikes.py. The pipeline is developed in Python, mainly using the numpy[1], scipy[2], cupy, and opencv packages, and consists of two main parts: a pre-processing that uses the current frame, the previous frame, and a mask of the region of interest; and a peak detection algorithm, which detects any peaks in the resulting pre-processed image. Prior to initiating the etSTED method and running the pipeline, a binary mask representing the region to consider in the FOV, usually the cell, is generated. It is created by an intensity thresholding and Gaussian smoothing of a mean image of several consecutive frames, usually 10. This mask will be input to any detection pipeline applied, and limits any uncorrelated background noise, which contains large ratiometric changes from frame to frame, from affecting the result.

In the analysis pipeline, the initial pre-processing transforms the widefield image into a map of pixelwise percentual intensity change from the previous image. It does so by first subtracting the previous frame from the current and dividing with the previous frame, generating a ratiometric image of pixelwise percentual change in intensity. This image is then multiplied by the pre-calculated mask to only get the changes inside the region of interest and discard the background. The image is then subjected to a Gaussian smoothing step to lower the impact of noise-based fluctuations of the fluorescence intensity changes, and the final pre-processed image is an intensity-insensitive map of the local changes in fluorescence, corresponding to the local changes in for example calcium level when using BAPTA-1. The intensity-insensitivity comes from looking at the ratiometric change of intensity rather than the raw intensity, and thus also small changes in calcium activity in dimmer local regions can be detected.

The subsequent peak detection uses a modified version of the peak detection algorithm used in *peak_local_max* in the scipy package. It takes the ratiometric image as input and compares it to a maximum filtered/dilated version of itself. The local maxima, and thus where we have intensity change peaks, are found as the coordinates where the two are equal. To avoid noise-based fluctuations of the fluorescent signal, such as Poisson and Gaussian noise, to affect our detection we multiply a Boolean mask with the intensity change peaks with a thresholded version of the ratiometric map. This absolute threshold on the intensity change ratio is perhaps the most important parameter of the analysis pipeline, and it must be adapted to the fluorescence signal level. Common values used in this work are between 0.05–0.15. Finally, peaks close to the border of the FOV are removed, to allow all the STED imaging to be performed inside the 80 x 80 μm^2 FOV. Then, the intensity values in the ratiometric image of the peaks that remain are extracted, and the brightest detected peaks are chosen, up to N peaks. The parameter N can be arbitrarily chosen in the widget and depends on how much information is wanted from the pipeline. While the triggered STED imaging will only be performed at the position of the

ratiometrically brightest peak in case of multiple detected peaks, post-acquisition analysis of the data could benefit from knowing where other detected peaks in the same frame were located.

Prior to return of the coordinates, the coordinates can additionally be sorted to ensure a provided spatial spacing between each one of them, to avoid multiple detections of the same peak. This comes at the cost of calculation speed, and as the current implementation of etSTED only performs STED imaging at the ratiometrically brightest peak and minimization of the analysis pipeline speed is of highest interest, this is rarely useful. One could imagine implementations of etSTED where multiple detected peak sites are investigated simultaneously or subsequently with STED imaging, and there this sorting of final detected coordinates could be useful.

The modification of the peak detection from the implementation in scipy is allowing most of the pipeline (all matrix operations included) to be run using the cupy package in Python and hence benefits from GPU-acceleration. For the size of widefield images used in this work, 800 × 800 pixels, this improves the runtime more than tenfold down to only 6 ms. This ultimately allows the method to start STED imaging inside < 40 ms from the point in time where any detected event occurs.

While the pipeline is designed and optimised to perform well in detection of calcium spikes in hippocampal neurons, it is also shown that it works well in HeLa cells. Moreover, as shown with pHluorin, the pipeline is generalizable to imaging of other fluorescent sensors with rapid intensity changes (< 50 ms for 20 Hz imaging) by adapting the pipeline parameters accordingly.

Supplementary Note 2. Real-time rising signal detection pipeline

A second analysis pipeline used in the work and optimised to detect more slowly rising signals over multiple frames, and often less bright, is dynamin_rise. It is developed to detect the rising signal in HeLa cells in widefield images of dynamin1-GFP, with a pixel size of 100 nm. Just as for the rapid_signal_spikes pipeline, this pipeline is used in the etSTED widget as a separate python function and uses the current widefield image, previous > 10 widefield images, and information from previous pipeline runs in the form of connected tracks of all previously localized spots, and outputs a detected event coordinate corresponding to a spot that has increased in intensity over the previous frames. The pipeline is available at https://github.com/jonatanalvelid/etSTED-widget/blob/main/analysis_pipelines/dynamin_rise.py. The pipeline is developed in Python and depends on the numpy, scipy, cupy, trackpy[3,4], and pandas[5,6] packages. It consists of three main steps: a pre-processing that uses only the current frame, and if provided a binary mask; a peak detection, selection, and connecting of tracks; and lastly a check of various threshold parameters from the input arguments on parameters of the tracks.

The current frame is initially smoothed using a Gaussian filter and thereafter a difference of Gaussians is applied in order to reduce uneven intensity background and noise in the image. Following this, the binary mask is applied, if provided, to only consider certain areas in the image and limit the effect of any uncorrelated background noise. A secondary step of Gaussian filter smoothing is additionally performed. After this preprocessing, the image is a noise-reduced version of the raw image, with the spots present in the raw widefield frame more clearly visualized.

The second step in the analysis pipeline is the peak detection. Here, similarly to rapid_signal_spikes, a modified version of the peak_local_max function from the scipy package

is used. This takes the preprocessed image as input and compares it to a maximum filtered version of itself. The local maxima are then found where the two images are equal. The peaks are put in a Boolean mask, and this mask is then multiplied with two thresholded versions of the preprocessed image: one with a high threshold and one with a low threshold. The low thresholding is performed in order to not consider any background noise that still might be present as peaks in the preprocessed image, and this parameter must be adjusted to the fluorescence signal level. The high thresholding is performed in order to remove any too bright peaks from consideration, that empirically appeared to affect the pipeline considerably by triggering many false events. The coordinates of the remaining peaks in the Boolean peak mask are retrieved, and then the peaks close to the border of the FOV are removed, to allow the STED imaging to be performed inside the 80 × 80 μm^2 FOV. The number of peaks used in the rest of the pipeline is then limited, using an input parameter, to reduce the processing time. The connection of tracks performed later is computationally heavy, and scales poorly with the number of peaks considered. In this work the maximum number of peaks used were around 150-200, a number however that was not always reached. Using faster CPUs or translating the linking algorithm to code running on the GPU are expected to improve this. The summed intensities of the surrounding areas of the remaining spots, in a 0.7 × 0.7 μm^2 square, are extracted. The information on the remaining spots, i.e. location, timepoint and intensity, are added to the pandas dataframe from the input arguments used to keep track of the tracks in the previously analysed frames. As the information from the current frame is added, information from the oldest frame previously considered is removed, to keep the considered timepoints to double that of the frames_appear input argument, which is for how many frames the intensity rise is considered. A common range of values used of frames_appear in this work is 5–8. A track linking algorithm from the trackpy package is then performed.

The last step in the pipeline is to compare parameters of the linked tracks to parameters inputted by the user. This is performed in four steps: (1) if a track appeared frames_appear ago, (2) if that track is detected for at least thresh_stayframes frames over the last frames_appear frames (commonly 70%), (3) if the intensity of that spot increases over thresh_stayframes with at least thresh_intincratio (commonly 1.06), and (4) if the track has not moved a too large vectorial distance (commonly 0.3 µm) in the last frames_appear. If a detected spot passes all four checks it is considered an event and the last coordinates of that track are returned as the event coordinates.

As with rapid_signal_spikes, the preprocessing and peak detection can run with GPU-acceleration by using the cupy package in Python, decreasing runtime as compared to running it all on the CPU. The connection of tracks for every frame is as mentioned the most time consuming part of the pipeline. In total, for the size of widefield images used in this work, 800 × 800 pixels, the pipeline runs in 20–60 ms, depending on the number of peaks present in the FOV and thus the number of tracks followed over time. As shown for rapid_signal_spikes, this pipeline is likely to work for detecting other similar events after parameter tweaking by the user. For example, if the event of interest involves a more slowly rising signal the frames_appear parameter could be increased.

Supplementary Note 3. Real-time vesicle proximity detection pipeline

A third pipeline developed as part of this work to detect the movement and proximity of vesicles is vesicle_proximity. It is optimised to identify potential interaction and fusion events between

intracellular vesicles in neurons in widefield images of CD63-GFP, with a pixel size of 100 nm. The pipeline is loaded and used in the etSTED widget as a separate python function and uses the current widefield image, previous > 10 widefield images, and information from previous pipeline runs in the form of connected tracks of all previously localized vesicles. It outputs a detected event coordinate corresponding to the location of a vesicle in which vicinity another vesicle has moved to and then disappeared from. The track disappearance is a sign that the two vesicles are very closely located in space, beyond the resolution of the widefield image, and thus can likely signify an interaction event. The pipeline is available at https://github.com/jonatanalvelid/etSTED-widget/blob/main/analysis_pipelines/vesicle_proximity.py. The pipeline is developed in Python and depends on the numpy, scipy, cupy, trackpy, and pandas packages. It consists of three main steps: a pre-processing using only the current frame and, if provided, a binary mask; a peak/vesicle localization, selection and connecting of tracks; and lastly a check of various threshold parameters from the user-provided input arguments on parameters of the tracks.

The pre-processing follows closely that of the dynamin_rise pipeline as the intermediate goal is the same: localize bright spots in the image after removing uneven background and noise. Thus, the pre-processing takes the current frame and performs a smoothing through Gaussian filtering, a difference of Gaussian step to reduce uneven intensity background and noise, a multiplication with the binary mask, and a secondary step of Gaussian filter smoothing. The intermediate image is a noise-reduced version of the raw widefield frame with the vesicles more clearly visualized.

The second step is similar to that both of the dynamin_rise and rapid_signal_spikes pipelines, with a peak detection as a modified version of the peak_local_max function from the scipy package. The detected peaks are filtered with a low threshold to not consider any background noise that still might be present. This parameter should be adjusted to the fluorescence signal level. The coordinates of the remaining peaks are retrieved, and the peaks close to the border of the FOV are removed to ensure STED imaging completely inside the $80 \times 80\ \mu m^2$ FOV. The number of peaks used in the rest of the pipeline is then limited to reduce the processing time, as for dynamin_rise. In this work the number of tracked vesicles is often limited to 200–250, however using faster CPUs or translating the linking algorithm to code running on the GPU is expected to increase that number. The location and timepoint of the remaining vesicles are then added to the pandas dataframe from the input arguments, used to keep track of the vesicles in the previously analysed frames. Information from the oldest frame is consequently removed to keep the considered timepoints to four times that of the stat_frames input argument, which is the threshold number of frames a vesicle track has to stay disappeared for after disappearing close to another vesicle. A commonly used value for stat_frames for intracellular vesicles in this work is 4.

The last step in the pipeline is to compare parameters of the linked vesicle tracks to parameters inputted by the user. This is performed as five individual check: (1) if a vesicle track disappeared stat_frames frames ago, (2) if another vesicle track is closer than ves_dist at the frame of disappearance (commonly 500 nm), (3) if both involved vesicle tracks have tracked points in a high ratio of frames leading up to the frame of disappearance (commonly 58%, to avoid noisy detections), (4) if at least one of the vesicles has moved an accumulated vectorial distance above a certain threshold (commonly 0.25 µm), and (5) if at least one of the vesicles has moved an accumulated absolute distance above a certain threshold from its starting position

(commonly 0.25 μm). If a tracked vesicle passes all five checks it is considered a possible interaction event and the last coordinates of the vesicle track still present are returned as the event coordinates.

As with the other pipelines, the pre-processing and peak detection can run with GPU-acceleration by using the cupy package in Python, decreasing runtime as compared to running it all on the CPU. The connection of tracks for every frame is the most time consuming part of the pipeline. In total for the size of widefield images used in this work, 800 × 800 pixels, the pipeline runs in 40–110 ms, depending on the number of vesicles present and tracked. The pipeline is likely to work for detecting events of proximity of other intracellular vesicles, such as lysosomes or peroxisomes, or other organelles, such as small mitochondria, after parameter tweaking by the user. For example, if the event of interest involves a more slowly moving entity the stat_frames parameter should be increased and the distance thresholds might have to be adjusted alongside it depending on the movement patterns.

Supplementary Note 4. Software implementation and generic control widget

The event-triggered imaging as presented here is an implementation of a general concept, hence not limited to neither the imaging techniques used; widefield and STED, neither to the software used for the presented imaging; ImSwitch (https://github.com/kasasxav/ImSwitch). Nevertheless, ImSwitch was important in the development of the method as it allows a fast and integrated control of multiple imaging modalities in a modular way, with minimal time delays. This allowed control of widefield imaging, STED imaging, and the development of a widget to control the event-triggered acquisition method. This implementation is ready to use in ImSwitch for any user, and as of now can be applied to the combination of widefield imaging with any scanning-based imaging technique.

To aid in the implementation and usage of this concept beyond ImSwitch, we provide a generic event-triggered imaging control software widget written in Python, based on the etSTED widget implemented in ImSwitch. The widget is provided in two versions; one base version (https://github.com/jonatanalvelid/etSTED-widget-base) and one version containing a simulated camera and image viewer (https://github.com/jonatanalvelid/etSTED-widget). Both versions are thoroughly commented and contains a connected widget and controller, both with the basic functionality implemented. The base version is ready to be implemented in other Python-based microscope control software by replacing the commented and marked code lines with software-specific function calls for basic control software functionality; follow the instructions in the readme. The simulation version shows this working by connecting the fast imaging function calls to the simulated camera and image viewer. Lastly, the base widget is executable and may be used as a standalone application if inter-software communication solutions are used. This has not been tested and might limit the temporal performance due to inter-software communication delays.

In the repositories there is an additional folder both for real-time analysis pipelines and coordinate transformation pipelines. See the readme for a full description of their use cases, as well as the Methods section for a full description of the analysis pipelines used throughout this work, as well as for a description on how the user can develop their own real-time analysis pipeline to be used in the widget.

Furthermore, Supplementary Note 5 denotes a generic recipe for implementation of event-triggered imaging on fast timescales in any microscope control software, of use for implementations when the provided widget solution cannot be directly adapted and used.

Supplementary Note 5. General event-triggered control implementation

The event-triggered imaging presented is based on a few concepts connected in a minimal and high-speed implementation. In the backend, this implementation works through eight main steps: (0) initiate event-triggered method; (1) record fast imaging image; (2) run analysis pipeline; (3) if detected coordinate: continue, if no detected coordinate: repeat from 1; (4) transform detected coordinate to scanning space; (5) prepare scan and calculate scanning curves; (6) run scan; and (7) save data and log file. Finally, there is a choice to repeat steps 1-7 in an endless loop for a fully automatic experiment acquisition of many events. In all of these steps, graphical elements allow the user to interact and adapt how the event-triggered imaging is running, and in such a way optimize and make sure that it is detecting the events of interest, and recording the scanned images of interest. In the GUI, the user can choose which laser and detector the fast method should use; which pipeline the method should use; set the editable parameters of the loaded pipeline; chose the coordinate transformation to use; and set the scanning parameters such as triggered image size, pixel size, dwell time, detectors and lasers.

It is helpful to allow the user to choose between different imaging modes such as experiments, validation or visualization. The experiment mode should run experiments with minimal graphical feedback for fastest performance and is the only mode that actually triggers scanning. Validation mode should trigger the recording of a number of additional fast imaging frames instead of scanning, allowing the user to check the performance in post-processing and prove that for example rapid signal spikes are in fact calcium signals that more slowly decrease after the trigger. Visualization mode should instead graphically show the pre-processed images that the analysis pipeline can return, and overlaid a scatter plot of the detected event coordinates, allowing the user to test out and optimize various sample or area-dependent pipeline parameters prior to running the real experiments.

Previous to the start of the experiment, the user can also perform a calibration of the coordinate transformation to use, in order to make sure that the scanning is performed in the exact same sample position as the event is detected at. Including this in the control widget allows easier calibration and thus always well-calibrated experiments. Additionally, the user can set threshold and smoothing parameters for a binary mask calculation, for use with pipelines that requires such a mask for proper functionality.

The implementation presented here uses a controller and widget solution, following the general MVP design pattern structure of ImSwitch. The widget contains all the GUI elements while the controller contains the logic, connections and control of the method. The controller should contain a set of functions that handles the eight steps of the acquisition process outlined above.

(0) requires a function that: reads the user-input pipeline parameter values from the GUI; resets general flags and parameters used throughout the event-triggered acquisition; checks which experiment mode the user has chosen; launches an additional image viewer if the experiment mode is validation or visualization; loads the coordinate transformation of choice; connects signals from the update of the fast imaging frame to running the pipeline and from the end of a scan to a function which takes care of resetting everything; reads the user-input scan parameters

from elsewhere in the control software; and in the end turns on the fast imaging laser. Following this, in step (1), a fast imaging frame is recorded through other parts of the software, and the imaging frame is updated in the image viewer of the software, alternatively directly sent to the event-triggered imaging widget through other forms of communication.

Then, (2) works through a function that starts to check a busy flag, and in case it is False continues and sets it to True. Following this, and throughout the functions, there are function calls to log the timestamps at various steps for the log file to be saved at the end. Then, the pipeline function is called with all the necessary parameters: current image, previous fast image frames, binary mask, run mode, extra information carried from earlier pipeline runs, and pipeline parameters.

After some additional check of flags such as run mode and not in the initial few frames, (3) is a check of if any coordinates of events were returned from the pipeline function call. If there is not, the current fast imaging frame is attached to a buffer of saved imaging frames that are used as input to the next function call and the function returns. If an event was detected, the fast imaging is paused, i.e. the signal from fast imaging frame update to run pipeline is disconnected and the fast imaging laser is turned off. The control then proceeds to (4). Here, the detected coordinate is rapidly transformed with the loaded coordinate transformation, and the returned coordinates in the scanning space is used to prepare the scanning and calculate the scanning curves in the next step.

Step (5) consists of setting the center coordinates of the previously loaded scanning parameters to the transformed event coordinate. These scan parameters are subsequently used to calculate scanning curves of the small ROI, which is performed in another part of the control software responsible for the image acquisition or scanning. In step (6), these scanning curves are used to call a signal or a function that executes the scanning with the defined parameters, once again in the image acquisition or scanning part of the software. Finally, when the scan has ended in (7), a function is called that saves the data from the experiment. Based on a user-defined variable then either the experiment is ended, by disconnecting the still connected signals from the end of a scan and resetting the parameters and flags used, or continued from step (1), by once again connecting signals from the update of a fast imaging frame to the pipeline running as well as turning on the fast laser.

The final saved data from step (7) includes not only the scanned image, but also a stack of the fast imaging frames leading up to the event, as well as a log file with for example the triggered event coordinates, the transformed coordinates, parameters for the analysis pipeline, and timestamps during different steps in the process. Additionally, for the validation mode, a number of fast imaging frames following the detected event are saved. The overall information allows full overview in post-acquisition analysis to understand, for example, if the triggering event was true and when in time the scanning took place with respect to the event.

The total event-triggered control can further be described using the pseudocode below, detailing the whole loop with the main function calls.

Pseudocode – event-triggered control

```
initiateExperiment()
frame = 0
while running:
  recordFastFrame()
  runAnalysisPipeline()
  if frame > init_frames:
    if visualizationMode or validationMode:
      graphicsUpdate()
      if validationMode:
        if validating:
          if post_event_frames > validation_frames:
            saveValidation()
            pauseFastModality()
            endRecording()
            continueFastModality()
          post_event_frames += 1
        elif event_detected:
          validating = True
          post_event_frames = 0
    elif event_detected:
      pauseFastModality()
      transform(coords_detected)
      initializeScan()
      runScan()
      graphicsUpdate()
      saveValidation()
      return
  bufferFastFrame()
  if validationMode:
    bufferPreprocessedFastFrame()
  frame += 1
```

Supplementary Note 6. Example etSTED experiments

We provide examples of etSTED experiments with full or more complete STED timelapses and the widefield of each triggering event in Extended Data Fig. 4, Extended Data Fig. 7–9, and Supplementary Fig. 5–6–6. Each experiment is performed in neurons or HeLa cells, and the STED imaging is performed on microtubules (SiR-tubulin, Supplementary Fig. 6), actin (SiR-actin, Supplementary Fig. 5), synaptic vesicles in active synapses (synaptotagmin-1_STAR635P, Extended Data Fig. 4), exocytosis membrane dynamics (Extended Data Fig. 8), endocytosis membrane dynamics (Extended Data Fig. 7), or intracellular vesicle interaction (Extended Data Fig. 9). All the recorded data in the experiments performed on these samples can further be found in the complementing shared dataset, see info under Data availability statement.

The experiment with etSTED imaging of microtubules (Supplementary Fig. 6) ran for 9 min 9 s and contained 16 detected events in total. Post-acquisition analysis determined 14 of them to be true events, and 2 false events caused by a moving filopodia. By investigating the triggering widefield frames and the calculated ratiometric images, 4 of the true events could be determined to be local calcium spikes in µm-sized limited regions such as single synapses, while the other

10 were found to be events with larger single or multiple neurite-wide signalling regions. The STED imaging was performed in regions of 5 × 5 μm^2 with a frame rate of 0.99 Hz for 30 frames. In the STED images we can see that the calcium events take place in regions and filaments rich of microtubule bundles. Thanks to the microtubules being present in almost all neurites and filaments, we can also confirm that the STED imaging takes place centered on the same area as the triggering event.

The experiment with etSTED imaging of actin (Supplementary Fig. 5) ran for 2 min 44 s and contained 12 detected events in total. Post-acquisition analysis determined 11 of them to be true events, and 1 false event caused by a moving filopodia. Of the true events, 4 could be determined to be local events, while the other 7 were found to be neurite-wide events. The STED imaging was performed in regions of 5 × 5 μm^2 with a frame rate of 0.99 Hz for 11 frames. In the STED images we see that the triggering calcium events happened in a mix of regions containing the membrane periodic skeleton, actin patches, and other structures.

The experiment with etSTED imaging of active synapses (Extended Data Fig. 4) ran for 3 min 9 s and contained 13 detected events in total. Post-acquisition analysis determined all of them to be true events. Of the true events, 9 could be determined to be local events, while the other 4 were found to be neurite-wide events. The STED imaging was performed in regions of 3 × 3 μm^2 with a frame rate of 2.46 Hz for 30 frames. In the STED timelapses we can follow the dynamics of the active synapses and presynaptic vesicles. In all events, we see active rearrangement of the clusters of presynaptic vesicles and movement of individual synaptic vesicles.

In Extended Data Fig. 7–10 there are examples of triggered STED imaging with respective triggering widefield frames, for STED imaging of exocytosis membrane dynamics, endocytosis membrane dynamics, and intracellular vesicle interaction. See Supplementary Table 1 and Supplementary Table 2 for further information on imaging conditions and analysis pipeline parameters for the visualized experimental data.

Supplementary Note 7. Post-acquisition true/false rapid signal spike event decision

To ensure that triggering events in the etSTED experiments performed with the *rapid_signal_spikes* pipeline throughout this work are true calcium spikes or CD63-pHluorin spike events, a decision-making pipeline has been performed post-acquisition. During the acquisition, not only the last widefield frame and the STED images are saved, but also the N widefield frames leading up to the event, in our case N = 10, are saved along with a log file describing the triggering event (coordinates, timings, etc.). These frames can be used in post-acquisition analysis to determine whether the triggering event was in fact a true event or caused by something else than an increase in the calcium signal. As the analysis pipeline detected differences in the fluorescence signal from one frame to the next inside each pixel, fluorescent details moving in the image, as well as other causes of a fast fluctuation in the fluorescence signal, can also trigger the STED imaging.

To sort out the false events, as further detailed in Supplementary Fig. 3, the N widefield frames before the event can be investigated. By using the saved triggering coordinate in the log-file, the summed signal trace surrounding the triggering coordinate, for example in a 5 × 5 μm^2 region, can be extracted up until the event was detected (Supplementary Fig. 3d). Any true event will show a significant increase in the last frame, and any false event due to something

moving fast in the frame to the detected pixel will show a relatively flat response. This is due to the fluorescence being present in surrounding pixels previously, assuming the object is not moving too fast for the 20 Hz widefield imaging to follow, and thus the movement is not causing any significant increase in the total fluorescence signal surrounding the affected pixel.

The real-time analysis pipeline could be improved performance-wise to not trigger on these events, by using the last few widefield frames instead of only the last to analyse the intensity traces as described above. However, this as any other added real-time analysis would cause a significant decrease in speed of the total analysis pipeline, leading to slower times between the actual event and the triggered STED images, and it is thus a trade-off and something that could be adjusted depending on the application at hand. We choose to minimize the number and complexity of the steps of the pipeline and thus optimise the speed, as the falsely triggered events can be sorted out in post-acquisition analysis as shown here. In our experiments with calcium spike detection in neurons or CD63-pHluorin spike detection in HeLa cells, moving filopodia are the most occurring false events and we have not detected any false triggering events other than moving fluorescent objects that can be sorted out in the way described above.

Supplementary Note 8. Temporal characterization of real-time analysis pipelines and etSTED experiments

We have characterized the temporal performance of example etSTED experiments, and the runtimes of the three real-time analysis pipelines developed and presented here. The characterization was performed using a sample of fluorescent beads with an approximate density of 0.03 μm^{-2} and moving the sample axially or laterally to trigger the different pipelines. The results of the characterization experiments are presented in Supplementary Table 3, for all analysis pipelines, and Supplementary Fig. 4, for the results on the *rapid_signal_spikes* pipeline, and includes various imaging conditions in terms of the processing unit used for running the analysis pipeline (CPU, GPU) and the size of the widefield image (200 × 200, 400 × 400, 800 × 800, 1000 × 1000, and 1500 × 1500 pixels).

All analysis pipelines show a clear decrease in computational time when partly implemented with GPU-acceleration as compared to purely running on the CPU. Additionally, all analysis pipelines show an increase in computational time when increasing the analysed image size. This increase in computational time scales differently with the image size depending on the pipeline. The *rapid_signal_spikes* pipeline scales directly with the amount of pixels, as it is based on the calculation of a ratiometric image. Instead, *dynamin_rise* and *vesicle_proximity* both includes a step of localization and tracking, and thus they scale directly with the number of tracked intensity peaks. This means that the *dynamin_rise* and *vesicle_proximity* pipeline runtimes might be shorter if the widefield image contains less tracked objects. Additionally, the *vesicle_proximity* pipeline heavily depends on parameter values provided, such as the distance threshold between a disappearing track and a present track marked as a potential event in the first check. The larger the distance threshold, the larger number of tracks to investigate and hence the longer runtime. This can be further understood from large disparity between the pipeline runtimes on the beads test sample, performed with different pipeline parameters, and the shorter runtimes from the experimental data on HeLa cells and neurons, for the *vesicle_proximity* pipeline. Instead, the *dynamin_rise* and *rapid_signal_spikes* pipelines show more consistent runtimes between test sample and experimental data.

As seen in Supplementary Fig. 4, the difference between runtime for the pipeline and time between widefield exposure start and STED scan start, i.e. overhead time, is constant and does not depend on the size of the widefield image. As expected, it neither depends on the analysis pipeline used (data not shown).

Supplementary Note 9. Manual STED timelapses of synaptotagmin-1

To compare the dynamics of the clusters of presynaptic vesicles in active synapses detected in the calcium-triggered STED images (Fig. 2g, Extended Data Fig. 4) to the dynamics without the presence of a calcium signal, we manually imaged STED timelapses of regions with the same size and frame rate and in the same samples, without considering the calcium signals (Extended Data Fig. 5a). The regions to image were chosen as regions with synaptotagmin-1 clusters in a confocal scan of the full 80 x 80 μm^2 field of view. To ensure that we were looking at similar clusters of presynaptic vesicles, we measured the area and aspect ratio of the largest cluster in each region of interest. The areas and aspect ratios of the clusters imaged in this way was not different to that of the clusters in the etSTED timelapses (Extended Data Fig. 5b). The mean area was 0.68 ± 0.63 μm^2 for the clusters in the calcium-triggered events, and 0.63 ± 0.52 μm^2 for the clusters in the manual timelapses. The aspect ratio was 2.3 ± 1.2 for the clusters in the calcium-triggered events, and 2.1 ± 0.7 for the clusters in the manual timelapses. Two-sided two-sample Kolmogorov-Smirnov tests performed on the distributions returned $p = 0.90$ (test statistic = 0.17) for the area, and $p = 0.76$ (test statistic = 0.20) for the aspect ratio.

Supplementary Note 10. Microscope components

The following is a list of the microscope components as labelled in Supplementary Fig. 2.

Abbreviations from Supplementary Fig. 2 and the list below: L, lens; SL, scan lens; TL, tube lens; OBJ, objective lens; BPF, band-pass filter; NF, notch filter; CUF, clean-up filter; DM, dichroic mirror; GX/GY, galvanometric mirror; F, fibre; FC, fibre coupler; PH, pinhole; AP, aperture; PBS, polarizing beam splitter; $\lambda/4$ / $\lambda/2$, wave plates; SM, spherical mirror; SLM, spatial light modulator; AOM, acousto-optic modulator; AOTF, acousto-optic tuneable filter; PSDB, picosecond delay box; DAQ, data acquisition system.

Lenses: L1: 300 mm, L2, L4, L15: 200 mm, L3: 100 mm, L5: 400 mm, L13: 150 mm (all AC254-XXX-B-ML, Thorlabs, Newton, USA), L6, L9: 100 mm, L7, L8: 200 mm, L10: 75 mm, L14: 30 mm (all AC254-XXX-A-ML, Thorlabs), L11: 250 mm, L12: 150 mm (both AC508-XXX-A-ML, Thorlabs), SL: 50 mm (Leica Microsystems, Wetzlar, Germany), TL: 200 mm (Leica Microsystems), OBJ: HC PL APO 100x/1.40 Oil STED White (15506378, Leica Microsystems). **Filters**: BPF1: ET705/100m (Chroma Technology, Bellows Falls, USA), BPF2: FF01-540/80 (Semrock, Rochester, USA), NF1: NF03-785E (Semrock), NF2 and NF3: ZET785NF (Chroma Technology), CUF: CT780/20bp (Chroma Technology). **Dichroic mirrors**: DM1: T700dcspxruv_UF3 (Chroma Technology), DM2: ZT405/488/561/640/775rpc (Chroma Technology), DM3: T860SPXRXT (Chroma Technology), DM4: FF552-Di02 (Semrock), DM5: Di02-R488 (Semrock). **Lasers**: 488: 06-MLD 488 nm (488 nm, Cobolt, Solna, Sweden), 640: LDH-D-C-640 (640 nm, PicoQuant, Berlin, Germany), 775: KATANA 08 HP (775 nm, OneFive, Regensdorf, Switzerland), 980: CP980S (Thorlabs). **Detectors/cameras**: APD: SPCM-AQRH-13-TR (Excelitas Technologies, Waltham, USA), WF-CMOS: ORCA-Flash4.0 v2 (Hamamatsu Photonics, Hamamatsu, Japan), FL-CMOS:

DMK 33UP1300 (The Imaging Source Europe, Bremen, Germany). **Scanners**: GX/GY: 6215H Galvanometric mirrors + 71215HHJ 671 Servo Driver (Cambridge Technology, Bedford, USA), Z-piezo: Z-piezo stage LT-Z-100 (Piezoconcept, Lyon, France), XY-stage: SCAN IM 130 × 85 – 2 mm (Märzhäuser Wetzlar, Wetzlar, Germany). **Fiber optics**: F1: PMJ-3AHPM3S-633-4/125-3-3-1 (OZ Optics), F2, F3: P5-488PM-FC-2 (Thorlabs), FC1, FC2: 60SMS-1-0-A2-02, FC3: 60SMS-1-0-A18-02, FC4: 60FC-4-M4-33 (all Schäfter + Kirchhoff, Hamburg, Germany). **Misc.**: PH: P75H (Thorlabs), AP: D15S (Thorlabs), PBS: PTW 1.15 (Bernard Halle Nachfl., Berlin, Germany), λ/4-1: 600-1200 achr. (RAC 5.4.15, Bernard Halle Nachfl.), λ/4-2: 460-680 achr. (RAC 3.4.15, Bernard Halle Nachfl.), λ/4-3 and λ/4-4: 500-900 achr. (RAC 4.4.15, Bernard Halle Nachfl.), λ/2: 500-900 achr. (RAC 4.2.15, Bernard Halle Nachfl.), SM: 50 mm concave mirror (CM508-050-P01, Thorlabs), SLM: LCOS-SLM X10468-02 (Hamamatsu Photonics), AOM: MT110-B50A1.5-IR-Hk + MDS1C-B65-34-85.135-RS (AA Opto Electronic, Orsay, France), AOTF: AOTFnC-400.650-TN + MPDS4C-B66-22-74.156 (AA Opto Electronic), PSDB: PSD-065-A-MOD (Micro Photon Devices, Bolzano, Italy), Mirrors: BB1-E02 and PF10-03-P01 (Thorlabs), Stand: DMi8 (Leica Microsystems). **PC:** CPU: Ryzen 7 3700X 8-core (AMD, Santa Clara, USA), GPU: GeForce RTX 3060 Ti TUF GAMING OC (ASUS, Taipei, Taiwan), DAQ: NI-DAQ PCIe-6353 (National Instruments, Austin, USA).

Supplementary figures

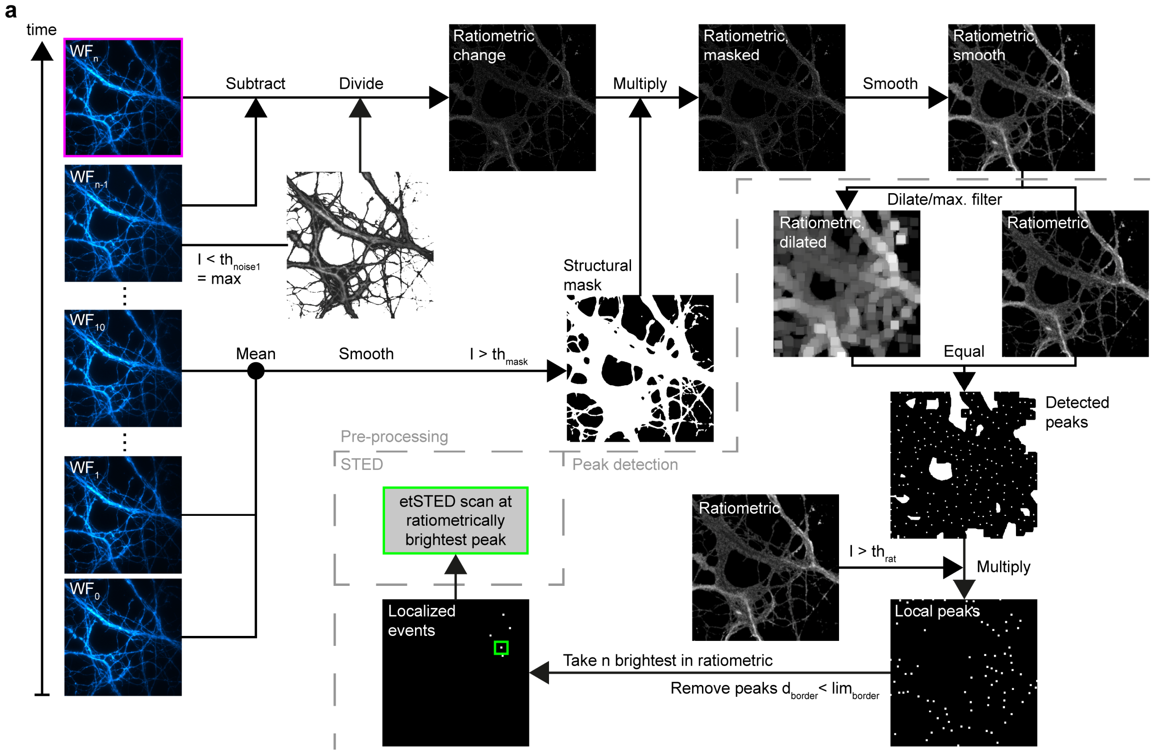

Supplementary Figure 1. Optimized rapid signal spikes event detection analysis pipeline. **a**, Schematic view of the analysis pipeline used to detect rapid signal spike events, such as those in BAPTA-1-labelled cells. The pipeline uses the current frame and compares to the previous frame in order to get an image of ratiometric pixel value changes. A binary mask of the area of interest, recorded in the beginning of an experiment, is multiplied with the ratiometric image, and further smoothing is applied. A peak detection is performed, and the detected peaks are further sorted to arrive at a potential detected event where STED imaging will be performed. See Supplementary Note 1 for a complete and thorough description of the different steps. th, threshold; max. filter, maximum filter; lim, limit; d, distance.

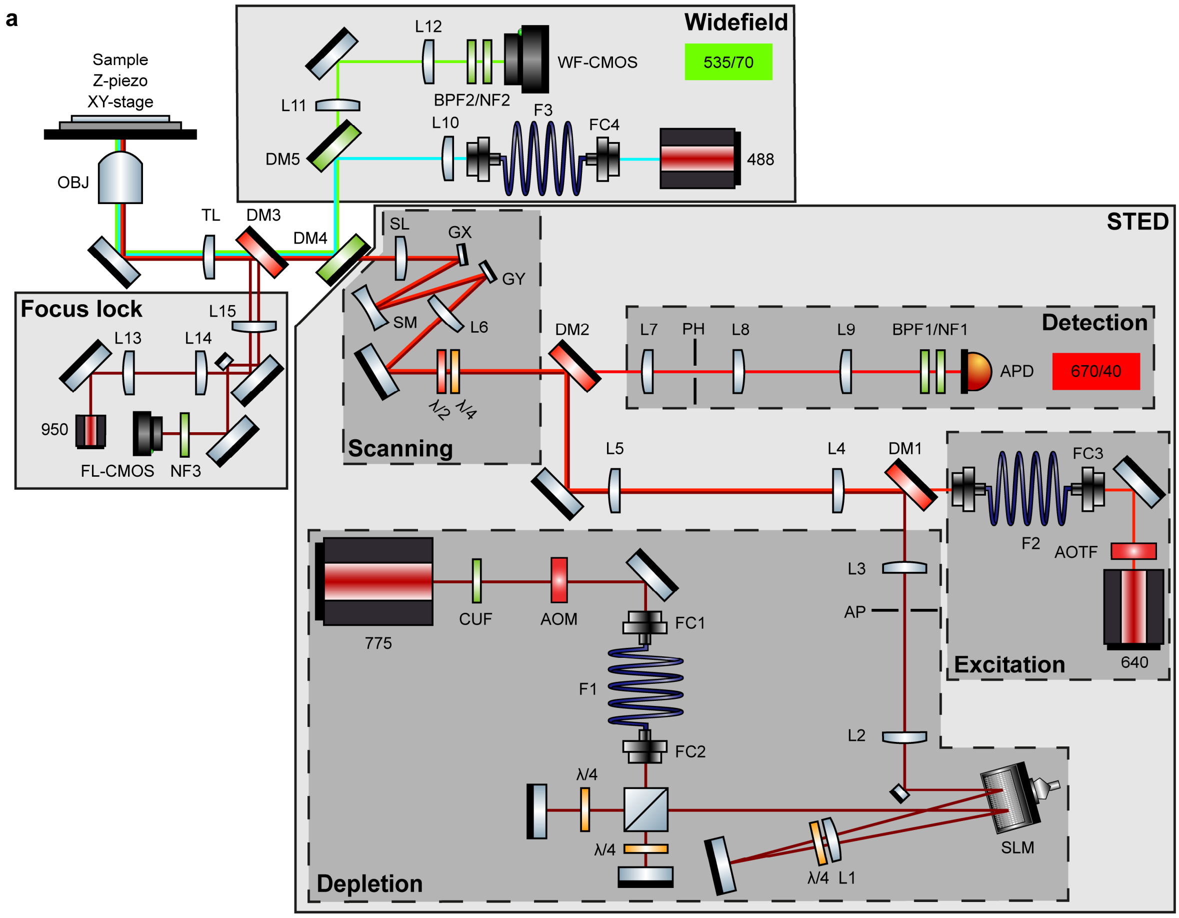

Supplementary Figure 2. etSTED setup. a, Extended schematic view of the etSTED setup, including boxes indicating the STED, widefield, and focus lock modules, as well as the excitation, depletion, scanning, and detection submodules of the STED module. The widefield module includes a CMOS camera and a fibre-coupled 488 nm excitation laser. The focus lock module includes a 950 nm infrared laser and a CMOS camera. The excitation submodule in the STED module contains a fibre-coupled 640 nm laser. The depletion submodule in the STED module contains a fibre-coupled 775 nm laser, an interferometric temporal polarization splitter, and a spatial light modulator. The scanning submodule in the STED module contains a dual-galvanometric-mirror scanning system with a spherical mirror. The detection submodule in the STED module contains a pinhole and a point-detector in the form of an APD. A detailed explanation of abbreviations and a list of all hardware models can be found in Supplementary Note 10.

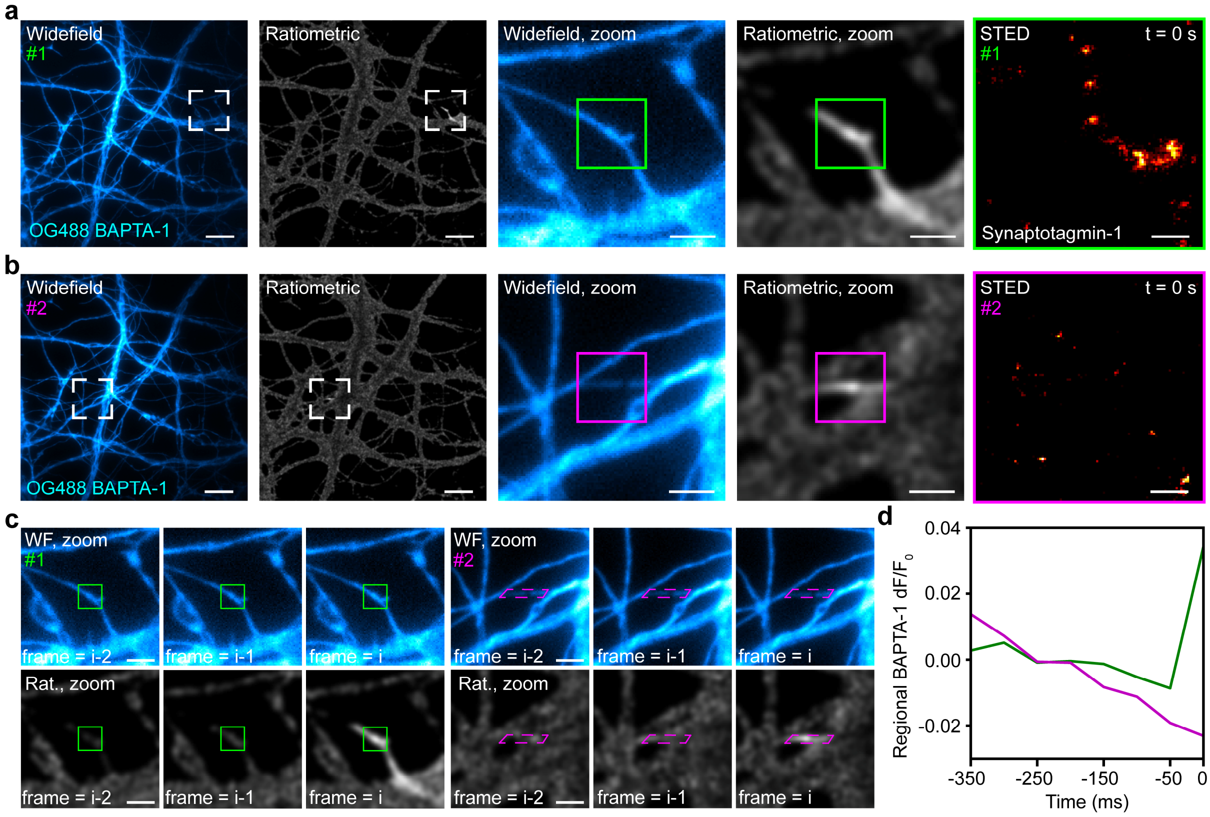

Supplementary Figure 3. Post-acquisition true/false detected event decision. a, Example widefield, ratiometric, and STED image from a true detected calcium event. White dashed boxes mark the zoomed-in regions (widefield, ratiometric). Green boxes mark the etSTED-imaged region (widefield, zoom; ratiometric, zoom). **b**, Example widefield, ratiometric, and STED image from a false detected calcium event. White dashed boxes mark the zoomed-in regions (widefield, ratiometric). Magenta boxes mark the etSTED-imaged region (widefield, zoom; ratiometric, zoom). **c**, Zoom-in of the widefield (top) and ratiometric (bottom) images for the three time points (frame = i-2, i-1, i) leading up to the detected event (frame = i), in the true (left) and false (right) event. Green boxes mark a region with increased calcium signal (true event). Magenta boxes mark a region with a moving filament (false event). **d**, Regional Oregon Green 488 BAPTA-1 fluorescence difference signal in a 5 × 5 μm^2 region around the detected event coordinate before the triggering event and in the triggering frame. Examples from a set of N = 186 events, N = 17 cells. See Supplementary Note 7 for a complete description of the procedure. Scale bars, 10 μm (**a**,**b** widefield, ratiometric), 2 μm (**a**,**b** zooms, **c**) and 500 nm (**a**,**b** STED).

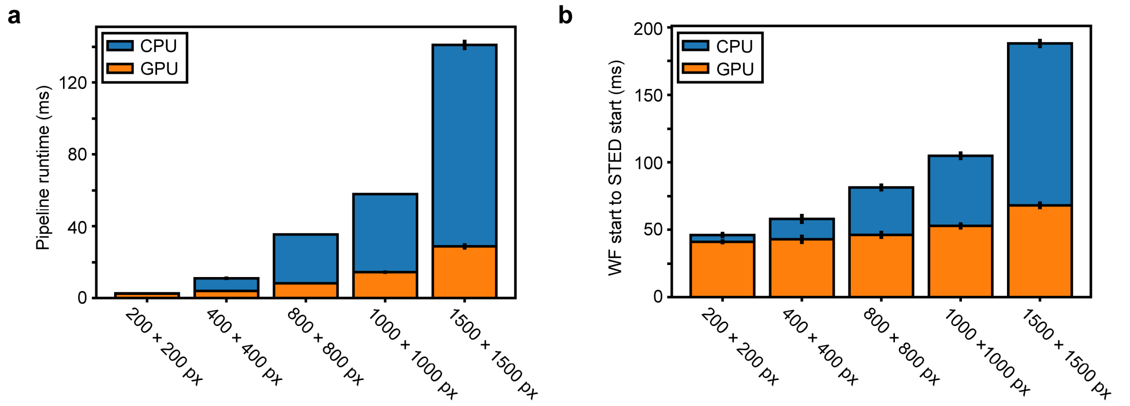

Supplementary Figure 4. Temporal characterization for etSTED experiments using the *rapid_signal_spikes* analysis pipeline. a, Pipeline runtime for *rapid_signal_spikes* in a test sample of fluorescent beads at a density of 0.03 μm^{-2} for different widefield image sizes (pixels) and using GPU-acceleration or only CPU. **b**, Total time between widefield exposure start to STED scan start at an event detection, for different imaging conditions as in **a**. Bars indicate mean and error bars indicate 1 standard deviation. N = 10 triggering events per condition.

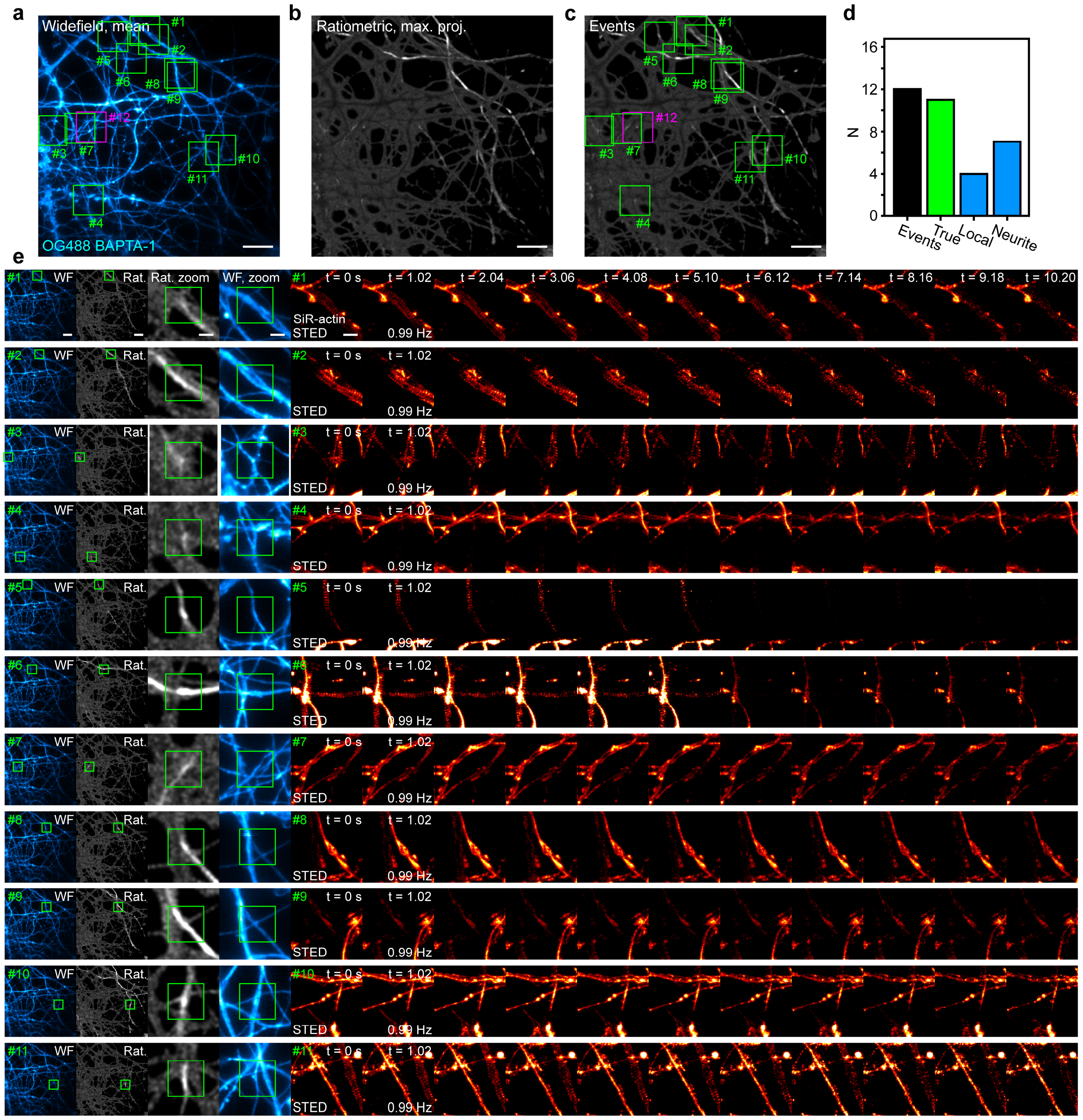

Supplementary Figure 5. etSTED experiment in neurons with calcium imaging (Oregon Green 488 BAPTA-1) and STED timelapse imaging of actin (SiR-actin). a, Mean image of all widefield frames with detected events. Boxes are centered on the coordinates of true (green) and false (magenta) detected events. **b**, Maximum projection of all ratiometric preprocessed widefield frames with detected events. **c**, Same as **b** with boxes centered on the coordinates of true (green) and false (magenta) detected events. **d**, Number of detected events, true events, local events, and neurite-wide events. **e**, Widefield frame, ratiometric preprocessed frame, zoom-ins of the widefield and ratiometric, and event-triggered STED timelapse (11 frames, 0.99 Hz) of the events as numbered and marked in **a**,**c**. N = 234 events, N = 19 cells. Boxes marks the center of the detected event. Same scales and time labels apply to all timelapses. Scale bars, 10 µm (**a**,**b**,**c**,**e** widefield and ratiometric), 2 µm (**e** zooms) and 1 µm (**e** STED).

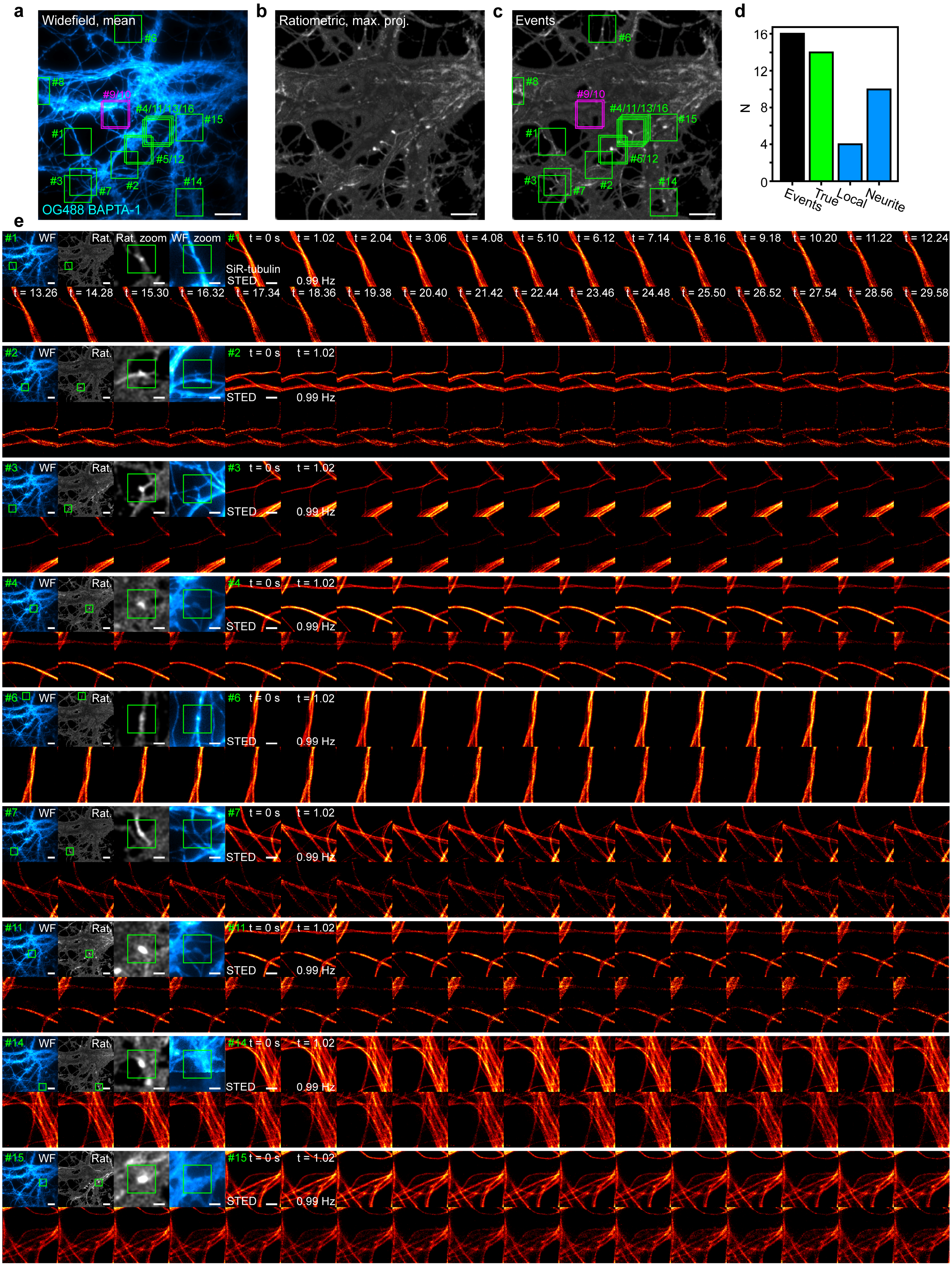

Supplementary Figure 6. etSTED experiment in neurons with calcium imaging (Oregon Green 488 BAPTA-1) and STED timelapse imaging of microtubules (SiR-tubulin). a, Mean image of all widefield frames with detected events. Boxes are centered on the coordinates of true (green) and false (magenta) detected events. **b**, Maximum projection of all ratiometric preprocessed widefield frames with detected events. **c**, Same as **b** with boxes centered on the

coordinates of true (green) and false (magenta) detected events. **d**, Number of detected events, true events, local events, and neurite-wide events. **e**, Widefield frame, ratiometric preprocessed frame, zoom-ins of the widefield and ratiometric, and event-triggered STED timelapse (30 frames, 0.99 Hz) of the events as numbered and marked in **a**,**c**. N = 147 events, N = 12 cells. Boxes marks the center of the detected event. Same scales and time labels apply to all timelapses. Scale bars, 10 µm (**a**,**b**,**c**,**e** widefield and ratiometric), 2 µm (**e** zooms) and 1 µm (**e** STED).

Supplementary references

1. Harris, C. R. *et al.* Array programming with NumPy. *Nature* **585**, 357–362 (2020).
2. Virtanen, P. *et al.* SciPy 1.0: fundamental algorithms for scientific computing in Python. *Nat. Methods* **17**, 261–272 (2020).
3. Allan, D. B., Caswell, T., Keim, N. C., van der Wel, C. M. & Verweij, R. W. *soft-matter/trackpy: Trackpy*. (2021).
4. Crocker, J. C. & Grier, D. G. Methods of Digital Video Microscopy for Colloidal Studies. *J. Colloid Interface Sci.* **179**, 298–310 (1996).
5. The pandas development team. *pandas-dev/pandas: Pandas*. (2020).
6. McKinney, W. Data Structures for Statistical Computing in Python. in 56–61 (2010). doi:10.25080/Majora-92bf1922-00a.

Supplementary tables

Supplementary Table 1. etSTED analysis pipeline parameters for the data presented in the figures.

Fig	min_ dist	thresh_ abs	num_ peaks	noise_ level	sm_ radius	ensure_ spacing	border_ lim	init_ sm				
**1a**	20	0.12	5	250	2	0	10	0				
**2a**	20	0.08	5	350	2	0	10	0				
**2h,i**	20	0.12	5	220	2	0	10	0				
**3h,i**	30	0.15	1	250	1	1	10	1				
**ED2**	20	0.06	5	700	2	0	10	0				
**ED3, S5**	20	0.12	5	250	2	0	10	0				
**ED4**	20	0.10	5	500	2	0	10	0				
**ED5**	20	0.12	5	220	2	0	10	0				
**ED6**	30	0.18	10	120	1	1	10	1				
**ED8**	30	0.15/0.17	1	250/400	1	1	10	1				
**S3**	20	0.12	5	220	2	0	10	0				
**S6**	20	0.12	5	500	2	0	10	0				
**Fig**	**min_ dist**	**num_ peaks**	**thresh_ abs_lo**	**thresh_ abs_hi**	**border_ lim**	**sm_ radius**	**mem_ frames**	**track_ se_dist**	**frame_ app**	**thresh_ st_rat**	**thresh_ int_rat**	**thresh_ move_d**
**3c,d**	4.0	150	40	400	10	0.7	5	4.0	3	0.7	1.05	3.0
**ED7**	4.0	150	35–55	300/400	10	0.7	5	4.0	3/8/10	0.7	1.05/1.06	3.0
**Fig**	**min_ dist**	**num_ peaks**	**thresh_ abs**	**border_ lim**	**sm_ radius**	**ves_ dist**	**stat_ frames**	**track_ se_dis**	**track_ mov_th**			
**4c,d**	4	100/150	300/550	10	0.7	7.0	5	4.0	2.5			
**ED9**	4	100/120	300/450	10	0.7	7.0	5	4.0	2.5			

Supplementary Table 2. etSTED sample and image acquisition parameters for widefield and STED for the data presented in the figures.

General			Widefield					STED						
**Figure**	**Sample**	**Labels**	**P_{488} (mW)**	**Img size (μm^2)**	**Px size (nm)**	**Exp time (ms)**	**Frame rate (Hz)**	**P_{640} (μW)**	**P_{775} (mW)**	**Img size (μm^2)**	**Px size (nm)**	**Dwell time (µs)**	**Frame rate (Hz)**	**Frames**
**1a**	Neurons	OG488-BAPTA-1, SiR-tubulin	0.31	80 × 80	100	20	20	5.0	73	3 × 3	30	30	-	-
**2a**	Neurons	OG488-BAPTA-1	0.35	80 × 80	100	50	20	-	-	-	-	-	-	-
**2h,i,j**	Neurons	OG488-BAPTA-1, Syt-1_STAR635P	0.31	80 × 80	100	20	20	12	59	3 × 3	30	30	2.5	31
**3c,d,e**	HeLa	Dynamin1-EGFP,Cholesterol_KK114	0.9	80 × 80	100	80	3.3	7	78<br>(3D)	3 × 3	30 × 60	30	5.9	50
**3h,i,j**	HeLa	CD63-pHluorin, Cholesterol_KK114	0.6	80 × 80	100	50	10	6	78<br>(3D)	1.5 × 3<br>1.8 × 3.6	30 × 60	30	11.2<br>8.0	50
**4c,d,e**	Neurons	CD63-EGFP, Sphingosyl-PE_KK114	0.6	80 × 80	100	30	5	6<br>8.5	124<br>78	3 × 3<br>2.5 × 2.5	30<br>25	30<br>50	2.7<br>1.6	31<br>21
**ED2**	HeLa	OG488-BAPTA-1, SiR-tubulin	1.90	80 × 80	100	100	10	8.5	61	2 × 2	25	30	-	-
**ED3, S5**	Neurons	OG488-BAPTA-1, SiR-actin	0.35	80 × 80	100	50	20	15	60	5 × 5	30	30	1.0	11
**ED4**	Neurons	OG488-BAPTA-1, Syt-1_STAR635P	0.31	80 × 80	100	20	20	12	59	3 × 3	30	30	2.5	31
**ED5**	Neurons	Synaptotagmin-1_STAR635P	-	-	-	-	-	12	59	3 × 3	30	30	2.5	31
**ED6**	Neurons	OG488-BAPTA-1, Syt-1_STAR635P	0.31	80 × 80	100	20	20	16	56–67	1 × 1	30	30	23.3	30
**ED7**	HeLa	Dynamin1-EGFP,Cholesterol_KK114	0.9–<br>1.2	80 × 80	100	80	3.3	6-7	78–97<br>(3D)	3 × 3<br>2 × 3<br>2 × 4	30 × 60	30	5.9<br>6.5<br>8.6	50
**ED8**	HeLa	CD63-pHluorin, Cholesterol_KK114	0.6	80 × 80	100	50	10	4.5–<br>6.5	78<br>(3D)	2 × 4<br>1.5 × 3	30 × 60<br>25 × 50	30	6.5<br>7.9<br>11.2	5<br>50
**ED9**	Neurons	CD63-EGFP, Sphingosyl-PE_KK114	0.9	80 × 80	100	30	5	6.5	111	2.5 × 2.5	30	40	2.9	31
**ED9**	Neurons	CD63-EGFP, Cholesterol_KK114	0.6	80 × 80	100	30	5	12	97	2.5 × 2.5	25	40	1.8	31
**S3**	Neurons	OG488-BAPTA-1, Syt-1_STAR635P	0.31	80 × 80	100	20	20	12	59	3 × 3	30	30	2.5	31
**S6**	Neurons	OG488-BAPTA-1, SiR-tubulin	0.30	80 × 80	100	20	20	5.0	73	5 × 5	30	30	1.0	31

Supplementary Table 3. Runtimes of etSTED analysis pipelines for CPU/GPU application and various image sizes. Measured on a sample of fluorescent beads (beads) or in HeLa or neuronal sample experiments (experiments). Gray background indicates cases used throughout the experiments presented in this work. N = 10 event detections per condition.

Pipeline	PU	Image (pixels)	Runtime, mean±std (ms)	Image (pixels)	Runtime, mean±std (ms)	Image (pixels)	Runtime, mean±std (ms)	Image (pixels)	Runtime, mean±std (ms)	Image (pixels)	Runtime, mean±std (ms)
**rapid_signal_spikes (beads)**	CPU	200 × 200	2.6 ± 0.5	400 × 400	11.2 ± 1.0	800 × 800	35.4 ± 0.7	1000 × 1000	58.0 ± 0.6	1500 × 1500	141 ± 3
**rapid_signal_spikes (beads)**	GPU	200 × 200	2.4 ± 0.5	400 × 400	3.9 ± 0.2	800 × 800	8.4 ± 0.5	1000 × 1000	14.6 ± 0.9	1500 × 1500	28.8 ± 1.7
**rapid_signal_spikes (experiments, syt-1)**	GPU	-	-	-	-	800 × 800	6.1 ± 0.4	-	-	-	-
**rapid_signal_spikes (experiments, pHl)**	GPU	-	-	-	-	800 × 800	10.1 ± 0.9	-	-	-	-
**dynamin_rise (beads)**	CPU	200 × 200	8.5 ± 0.5	400 × 400	22.4 ± 0.7	800 × 800	55.1 ± 0.8	1000 × 1000	76.5 ± 0.9	1500 × 1500	164 ± 2
**dynamin_rise (beads)**	GPU	200 × 200	9.9 ± 0.5	400 × 400	16.3 ± 2.5	800 × 800	27.3 ± 4.6	1000 × 1000	35.7 ± 19.7	1500 × 1500	40.5 ± 1.8
**dynamin_rise (experiments, dyn)**	GPU	-	-	-	-	800 × 800	25.6 ± 11.2	-	-	-	-
**vesicle_proximity (beads)**	CPU	200 × 200	112 ± 78	400 × 400	154 ± 19	800 × 800	375 ± 219	1000 × 1000	548 ± 286	-	-
**vesicle_proximity (beads)**	GPU	200 × 200	16.0 ± 4.8	400 × 400	28.6 ± 3.1	800 × 800	212 ± 244	1000 × 1000	337 ± 215	-	-
**vesicle_proximity (experiments, cd63)**	GPU	-	-	-	-	800 × 800	61.2 ± 30.5	-	-	-	-
