# Supplementary material for: Event-triggered STED imaging
Source: Nat Methods. 2022 Sep 8;19(10):1268–75. doi: 10.1038/s41592-022-01588-y (PMC9550628; doi:10.1038/s41592-022-01588-y)
Supplement: Supplementary file 2 — Reporting Summary [file 41592_2022_1588_MOESM2_ESM.pdf]

nature portfolio

Corresponding author(s): Ilaria Testa

Last updated by author(s): Jun 22, 2022

Reporting Summary

Nature Portfolio wishes to improve the reproducibility of the work that we publish. This form provides structure for consistency and transparency in reporting. For further information on Nature Portfolio policies, see our Editorial Policies and the Editorial Policy Checklist.

Statistics

For all statistical analyses, confirm that the following items are present in the figure legend, table legend, main text, or Methods section.

n/a	Confirmed	
☐	☒	The exact sample size (*n*) for each experimental group/condition, given as a discrete number and unit of measurement
☐	☒	A statement on whether measurements were taken from distinct samples or whether the same sample was measured repeatedly
☐	☒	The statistical test(s) used AND whether they are one- or two-sided<br>*Only common tests should be described solely by name; describe more complex techniques in the Methods section.*
☒	☐	A description of all covariates tested
☒	☐	A description of any assumptions or corrections, such as tests of normality and adjustment for multiple comparisons
☐	☒	A full description of the statistical parameters including central tendency (e.g. means) or other basic estimates (e.g. regression coefficient) AND variation (e.g. standard deviation) or associated estimates of uncertainty (e.g. confidence intervals)
☐	☒	For null hypothesis testing, the test statistic (e.g. *F*, *t*, *r*) with confidence intervals, effect sizes, degrees of freedom and *P* value noted<br>*Give P values as exact values whenever suitable.*
☒	☐	For Bayesian analysis, information on the choice of priors and Markov chain Monte Carlo settings
☒	☐	For hierarchical and complex designs, identification of the appropriate level for tests and full reporting of outcomes
☒	☐	Estimates of effect sizes (e.g. Cohen's *d*, Pearson's *r*), indicating how they were calculated

Our web collection on statistics for biologists contains articles on many of the points above.

Software and code

Policy information about availability of computer code

Data collection	The imaging was performed with open-source hardware and acquisition control software ImSwitch in Python, developed in the lab and available at https://github.com/kasasxav/ImSwitch (v1.3.0) and https://github.com/jonatanalvelid/ImSwitch-etSTED (v1.0). The etSTED acquisition method was implemented as a widget in ImSwitch, available as the ImSwitch-adapted widget at https://github.com/etSTED-widget (v1.0), as a standalone software-agnostic widget at https://github.com/jonatanalvelid/etSTED-widget-base (v1.0), and in the main ImSwitch repository.
Data analysis	Real-time analysis and post-acquisition data analysis was performed with custom-written scripts and JupyterLab notebooks in Fiji (ImageJ v1.53c) and Python (v3.7-v3.9). Overall, the control software and analysis was performed with the following Python packages: numpy (v1.19), scipy (v1.6), scikit-image (v0.18), cupy-cuda100 (v9.0.0), matplotlib (v3.4.1), opencv (v4.5.2.54), trackpy (v0.5.0), pandas (v1.3.2), pyqtgraph (v0.12.3), napari (v0.4.7), and jupyterlab (v3.2.9). For deconvolution, the Imspector software (Max-Planck Innovation) was used.<br><br>Post-acquisition analysis scripts are available at https://github.com/jonatanalvelid/etSTEDanalysis. Real-time image analysis during etSTED acquisition was performed using three optimized image analysis pipelines: rapid_signal_spikes (https://github.com/jonatanalvelid/etSTED-widget/blob/main/analysis_pipelines/rapid_signal_spikes.py), dynamin_rise (https://github.com/jonatanalvelid/etSTED-widget/blob/main/analysis_pipelines/dynamin_rise.py), and vesicle_proximity (https://github.com/jonatanalvelid/etSTED-widget/blob/main/analysis_pipelines/vesicle_proximity.py).

For manuscripts utilizing custom algorithms or software that are central to the research but not yet described in published literature, software must be made available to editors and reviewers. We strongly encourage code deposition in a community repository (e.g. GitHub). See the Nature Portfolio guidelines for submitting code & software for further information.

Data

Policy information about availability of data

All manuscripts must include a data availability statement. This statement should provide the following information, where applicable:

- Accession codes, unique identifiers, or web links for publicly available datasets
- A description of any restrictions on data availability
- For clinical datasets or third party data, please ensure that the statement adheres to our policy

The data that support the implementation of the method and support the findings in this study, including images, log-files, and metadata, are openly available in Zenodo at https://doi.org/10.5281/zenodo.5593270, reference number 5593270.

Field-specific reporting

Please select the one below that is the best fit for your research. If you are not sure, read the appropriate sections before making your selection.

☒ Life sciences ☐ Behavioural & social sciences ☐ Ecological, evolutionary & environmental sciences

For a reference copy of the document with all sections, see nature.com/documents/nr-reporting-summary-flat.pdf

Life sciences study design

All studies must disclose on these points even when the disclosure is negative.

Sample size	No sample size dependent statistical testing was performed. Sample sizes for the different experiments were chosen to multiple experimental days and cover glass replications (N = 2–7), many cell replications on each glass (N = 2–9, N = 8–62 cells in total), and on average many triggered or manual images/timelapses/events in each cell (N = 2–37, N = 14–379 events in total); see figure legends for specific numbers for each experiment. This ensures a large enough statistical ground during statistical testing, and was chosen in order to make sure to contain recorded images with similar image quality and resolution independently from the cell, glass or experiment.
Data exclusions	No data was excluded from the analysis.
Replication	The STED imaging quality was reproducible in different cells, different days of recording and for extended times after system alignment. System alignment was stable over time for the full day of recording. Experiments were replicated and performed independently multiple times inside a time period of half a year. All attempts at replication were successful.
Randomization	No allocation into experimental groups was performed.
Blinding	No allocation into experimental groups was performed.

Reporting for specific materials, systems and methods

We require information from authors about some types of materials, experimental systems and methods used in many studies. Here, indicate whether each material, system or method listed is relevant to your study. If you are not sure if a list item applies to your research, read the appropriate section before selecting a response.

Materials & experimental systems

n/a	Involved in the study	
☐	☒	Antibodies
☐	☒	Eukaryotic cell lines
☒	☐	Palaeontology and archaeology
☐	☒	Animals and other organisms
☒	☐	Human research participants
☒	☐	Clinical data
☒	☐	Dual use research of concern

Methods

n/a	Involved in the study	
☒	☐	ChIP-seq
☒	☐	Flow cytometry
☒	☐	MRI-based neuroimaging

Antibodies

Antibodies used	Synaptotagmin-1 antibody luminal domain (Synaptic Systems, cat. no. 105 3FB); FluoTag-X2 anti-mouse Ig kappa light chain nanobody conjugated to Abberior STAR635P (NanoTag Biotechnologies, cat. no. N1202-Ab635P).
Validation	The antibody against synaptotagmin-1 has been tested and used in many publications over the past 20 years. Here are a few examples: (1) Geppert M., Annual review of neuroscience (1998) 21: 75-95, (2) Jahn R., Annual review of neuroscience (1994) 17:

March 2021

219-46, (3) Südhof TC, Nature (1995) 3756533: 645-53. We think that a formal validation is not necessary in this case.

Eukaryotic cell lines

Policy information about cell lines

Cell line source(s)	HeLa, ATCC CCL-2
Authentication	None of the cell lines used were authenticated.
Mycoplasma contamination	No testing for mycoplasma contamination.
Commonly misidentified lines (See ICLAC register)	No commonly misidentified cell lines were used in this study.

Animals and other organisms

Policy information about studies involving animals; ARRIVE guidelines recommended for reporting animal research

Laboratory animals	Sprague Dawley rat, embryonic day 18
Wild animals	The study did not involve wild animals.
Field-collected samples	The study did not involve field-collected samples.
Ethics oversight	All experiments were performed in accordance with animal welfare guidelines set forth by Karolinska Institutet and were approved by Stockholm North Ethical Evaluation Board for Animal Research.

Note that full information on the approval of the study protocol must also be provided in the manuscript.
